# Medical knowledge of ChatGPT in public health, infectious diseases, COVID-19 pandemic, and vaccines: multiple choice questions examination based performance

**DOI:** 10.3389/fpubh.2024.1360597

**Published:** 2024-04-17

**Authors:** Sultan Ayoub Meo, Metib Alotaibi, Muhammad Zain Sultan Meo, Muhammad Omair Sultan Meo, Mashhood Hamid

**Affiliations:** ^1^Department of Physiology, College of Medicine, King Saud University, Riyadh, Saudi Arabia; ^2^Department of Medicine, College of Medicine, King Saud University, Riyadh, Saudi Arabia; ^3^College of Medicine, Alfaisal University, Riyadh, Saudi Arabia; ^4^Department of Family and Community Medicine, College of Medicine, King Saud University, Riyadh, Saudi Arabia

**Keywords:** ChatGPT, artificial intelligence, knowledge, public health, infectious disease, medical education

## Abstract

**Background:**

At the beginning of the year 2023, the Chatbot Generative Pre-Trained Transformer (ChatGPT) gained remarkable attention from the public. There is a great discussion about ChatGPT and its knowledge in medical sciences, however, literature is lacking to evaluate the ChatGPT knowledge level in public health. Therefore, this study investigates the knowledge of ChatGPT in public health, infectious diseases, the COVID-19 pandemic, and its vaccines.

**Methods:**

Multiple Choice Questions (MCQs) bank was established. The question’s contents were reviewed and confirmed that the questions were appropriate to the contents. The MCQs were based on the case scenario, with four sub-stems, with a single correct answer. From the MCQs bank, 60 MCQs we selected, 30 MCQs were from public health, and infectious diseases topics, 17 MCQs were from the COVID-19 pandemic, and 13 MCQs were on COVID-19 vaccines. Each MCQ was manually entered, and tasks were given to determine the knowledge level of ChatGPT on MCQs.

**Results:**

Out of a total of 60 MCQs in public health, infectious diseases, the COVID-19 pandemic, and vaccines, ChatGPT attempted all the MCQs and obtained 17/30 (56.66%) marks in public health, infectious diseases, 15/17 (88.23%) in COVID-19, and 12/13 (92.30%) marks in COVID-19 vaccines MCQs, with an overall score of 44/60 (73.33%). The observed results of the correct answers in each section were significantly higher (*p* = 0.001). The ChatGPT obtained satisfactory grades in all three domains of public health, infectious diseases, and COVID-19 pandemic-allied examination.

**Conclusion:**

ChatGPT has satisfactory knowledge of public health, infectious diseases, the COVID-19 pandemic, and its vaccines. In future, ChatGPT may assist medical educators, academicians, and healthcare professionals in providing a better understanding of public health, infectious diseases, the COVID-19 pandemic, and vaccines.

## 1 Introduction

The “Chatbot Generative Pre-Trained Transformer (ChatGPT), developed by OpenAI is an Artificial Intelligence (AI)” based tool that generates dialogs with humans. ChatGPT is developed with highly advanced technology to answer users’ questions (1), swiftly clarify and provide the pleaded information. It is a valuable tool for getting ideas on any topic, article writing, and information searching for academic execution (2). At the beginning of 2023, ChatGPT received great attention among academicians and researchers as it offered attractive information for students, faculty, and educators. This is also a fact that it poses threats to the traditional structure of research and educational systems, and it may increase the chances of cheating on online exams and may minimize human rational, and cognitive abilities (3).

ChatGPT has shown impressive performance in various domains, including academia, research, and creative writing. However, challenges and concerns related to biases and trust persist (4). The science community and public views exist with some level of limitations about the adequacy and usage of ChatGPT in academia, research, and healthcare (5, 6). ChatGPT has the potential to enhance learning and connect the stakeholders in academic and research settings. The potential of ChatGPT is not limited to the development of personalized and complex learning, assessments, and accuracy of the information. Still, there are several challenges that ChatGPT is facing in education. The problems linked with plagiarism, misuse or lack of knowledge, accountability, academic integrity, privacy, ethics, and professionalism (6). The controversies about ChatGPT and its implications in higher education are the major limitations to the implementation of ChatGPT in academia (7).

There is a great discussion about ChatGPT and its knowledge, but the literature is lacking in determining the knowledge level of ChatGPT in public health and infectious disease topics such as the COVID-19 pandemic and its vaccines. The “Severe Acute Respiratory Syndrome Coronavirus 2 (SARS-CoV-2)” also known as the COVID-19 pandemic is a highly challenging issue and a topic of great interest to the public, the science community, and policymakers (8). Worldwide, the pandemic has claimed the lives of over seven million people and has disturbed the economies, education systems, and daily lives (9). While major progress has been made in developing COVID-19 vaccines, but still the threat of new variants and the uneven distribution of these medical resources still pose continued challenges (10). The pandemic has affected marginalized communities, including low-income nations, racial and ethnic minorities, and those with pre-existing health conditions (11). In this situation, it is important to understand the need for equitable distribution of healthcare resources, improved accessibility to testing and treatment, and the prioritization of vulnerable populations in vaccination campaigns (12). The global situation due to the pandemic has exposed the global healthcare systems and the interconnectedness of the world. The COVID-19 pandemic has challenged global societies in unprecedented ways, and its impact on public health is highly essential to understand to combat such a global crisis (13).

The COVID-19 vaccines play a crucial role in controlling the coronavirus’s spread and mitigating the pandemic’s impact. The COVID-19 vaccines are incredibly important in the prevention of the COVID-19 pandemic, provide protection against the SARS-CoV-2 virus, reduction in hospitalizations and deaths. The people who are vaccinated are less likely to experience severe illness, and need hospitalization, and vaccines have demonstrated a highly effective in preventing severe cases and reducing the risk of mortality. It develops herd immunity, resumption of normalcy, protects vulnerable populations, reduces transmission, vaccines protect these vulnerable groups and decreases the risk of exposure, and limits virus mutations (14).

The MCQ pattern of examinations is important in assessing the knowledge in numerous disciplines of medical sciences (15). MCQs are well-acknowledged and widely used tools in medical education examinations which can assess the higher levels of learning strategies (16, 17). Worldwide, various institutes are using the MCQs in medical sciences examinations to assess knowledge and skills. The MCQs help in better conceptual understanding developing clinical reasoning skills and better tools in medical and health care institutes (18, 19). The case scenario-based MCQs assess higher-order cognitive skills (20). MCQs are better at assessing higher cognitive skills and critical thinking skills, deeper conceptual understanding, and evaluation of higher cognitive functions (21, 22). At the beginning of the year 2023, worldwide ChatGPT received great attention for its role among students, faculty, academicians, and researchers. However, this is also a fact that with any technology including ChatGPT, there are some limitations and challenges to consider while using ChatGPT in educational settings. This study hypothesis proposes that ChatGPT may have the medical knowledge to achieve the appropriate grades in public health, infectious disease, and COVID-19 vaccines MCQ-based assessments. Therefore, the present study aimed to investigate the medical knowledge of ChatGPT in infectious diseases, the COVID-19 pandemic, and vaccines on multiple choice questions (MCQs) examination-based performance.

## 2 Research methodology

### 2.1 Multiple choice questions examination bank

In this study, the research team members established a Multiple-Choice Questions (MCQs) bank based on the subject information from “First Aid USMLE Step1; AMBOSS Step 1, Harrison’s infectious disease book, Climate and COVID-19 pandemic book, Vaccines Fact Sheet, World Health Organization, Centers for Disease Control and Prevention, Food and Drug Authorities USA, Elsevier COVID-19 Resource Centre, and the university examination pools.” After the establishment of the MCQs questions bank pool, the investigators examined all the question contents and were satisfied that the MCQs were related to the subject contents. The questions were based on the case scenario, with four sub-stems and had a single correct answer.

While establishing the MCQs bank, the types and difficulty levels of MCQs were checked, which is important for confirming the quality and accurate assessments of knowledge. Two senior faculty with a medical education background reviewed all the MCQs and analyzed the clarity, accuracy, and alignment with the contents. Moreover, any distractors and potential biases were also checked. The difficulty level, clarity, and potential test-taking issues were also evaluated. The complex wording, ambiguous language, or technical jargon were minimized. Based on these factors, the difficulty level of MCQs was examined to make the assessment more accurate, reliable, and valid. The research team was satisfied with the required standard of MCQs, and after that, the MCQs were transferred to the MCQs pool. The pattern was based on the questions required in medical education, such as, “The patient’s condition is most probably caused by which of the following pathogens? What is the most appropriate dose of the COVID-19 vaccine? Which one of the following temperatures is required for the storage of the vaccine.” The questions were scored according to the number of correct answers. The MCQs were cautiously checked for the related content, main stem, sub-stems, and answer keys. Any MCQ that had images were excluded from the study. We also excluded 03 MCQs from the pool which were used for the technical checking. After the establishment of the MCQs bank, we obtained 60 MCQs, 30 MCQs from infectious diseases topics, 17 MCQs from the COVID-19 pandemic, and 13 MCQs on COVID-19 vaccines. The tasks were given to evaluate the knowledge level of ChatGPT on MCQs.

### 2.2 ChatGPT response rate collection

The ChatGPT response rate on the MCQs was obtained between July 2, 2023, to September 20, 2023. The research team members manually entered MCQ one by one MCQ, and a new ChatGPT session was started for each entry to evade information-retaining prejudice. On each entry of MCQ, ChatGPT provided the answers with explanations. The first response of ChatGPT was considered final, and no attempt was made as a choice of “regenerate response.” As per the established answer key, grades were calculated on a scale of 0–1, zero representing incorrect and one representing correct answer.

### 2.3 Statistical analysis

The data were analyzed using descriptive statistical tests and expressed as numbers and percentages. The comparison between right and wrong MCQs was assessed. The *p*-values were calculated using the binomial test on Statistical Package for the Social Sciences (SPSS 29.0.1.0). The binomial test is useful as it deals with categorical data where there are possible outcomes such as one MCQS was right or three were wrong. To calculate a *p*-value using the binomial test, the steps followed were to formulate hypotheses, choose the significance level, count the number of right and wrong, and calculate the probability. This is done using the binomial probability formula. Since all the MCQs had four options, this made the chance of getting the question right 25%. A calculated *p*-value of less than 0.05 was considered statistically significant.

## 3 Results

The ChatGPT knowledge was assessed on the individual MCQs in public health, infectious diseases, the COVID-19 pandemic, and vaccines. The MCQs were comprehensive, highly standardized questions that covered assorted topics with basic, clinical concepts and facts in the field of public health and infectious diseases, the COVID-19 pandemic, and vaccines. A total of 60 Multiple Choice Questions (MCQs) on infectious diseases (30 MCQs) the COVID-19 pandemic (17 MCQs) and vaccines (13 MCQs) were randomly selected, prepared based on the information available in various national, and international examination pools, textbooks, facts sheets and the task were given to evaluate the ChatGPT knowledge level (Table 1).

**Table 1:** Distribution of MCQs about public health, infectious diseases, the COVID-19 pandemic, and vaccines.

Distribution of MCQs	Number of MCQs
Public health, infectious diseases	30
COVID 19 pandemic	17
COVID 19 vaccines	13
Total MCQs	60

**Figure 1:**
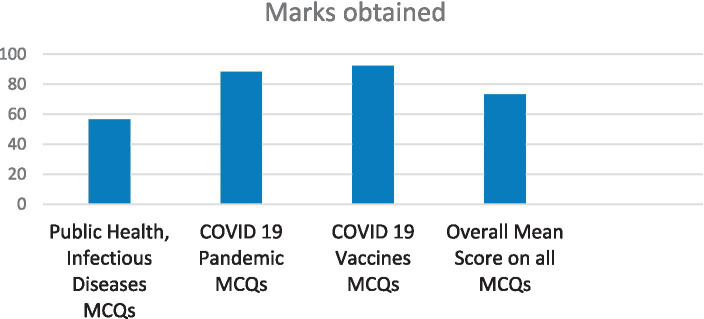
Marks obtained by ChatGPT in public health, infectious diseases, and COVID-19 pandemic and vaccines examination.

Table 2 demonstrates the knowledge level of ChatGPT on the established set of MCQs. The grades achieved on MCQs are shown in Table 2. Out of 60 MCQs in public health, infectious diseases, the COVID-19 pandemic, and vaccines, ChatGPT attained 17/30 (56.66%) marks in infectious diseases MCQs, 15/17 (88.23%) in COVID-19 MCQs, and 12/13 (92.30%) marks in COVID-19 allied Vaccines MCQs, with an overall score of 44/60 (73.33%) (Table 2; Figure 1).

**Table 2:** Marks obtained by ChatGPT on MCQs in public health, infectious diseases, COVID-19 pandemic, and its vaccines.

MCQs	Marks obtained (%)	Significance level
Public health, infectious diseases	17/30 (56.66%)	*p* = 0.001
COVID 19 pandemic	15/17 (88.23%)	*p* = 0.001
COVID 19 vaccines	12 /13 (92.30%)	*p* = 0.001
Total MCQs	44 / 60 (73.33%)	*p* = 0.001

The analysis further showed that the *p*-values were less than (*p* = 0.05), indicating that the observed results of the correct answers in each section were significantly higher than what would be expected by chance alone if the answers were guessed at random (Table 2).

ChatGPT obtained a satisfactory score in all three domains of COVID-19 pandemic exams with no focused instruction or guided support. These results highlight the role of ChatGPT, it may assist health professionals and policymakers in assisting such pandemic-allied situations.

## 4 Discussion

ChatGPT is a novel accomplishment of this most modern and technology-based highly advanced world. It achieved great attention globally as it rapidly responds to questions and provides appropriate answers on the required topic. It is useful for enhancing knowledge, providing explanations, offering suggestions, creating conversational dialogs, and assisting with multiple tasks (23). Worldwide, people are searching for updated knowledge of the COVID-19 pandemic hence the use of ChatGPT has increased as information information-providing tool. However, the literature is lacking on whether ChatGPT provides correct information or not. Therefore, the present study investigated the ChatGPT knowledge in public health, infectious diseases, the COVID-19 pandemic, and its vaccines. The present study findings reveal that ChatGPT achieved a good grade in MCQs-based examination in public health, infectious diseases, the COVID-19 pandemic, and its allied vaccines. The analytic thinking approach in medical education is fundamental to producing proficient physicians. Hence, at regional and international levels, medical educationalists and examination bodies introduced MCQ-based examination systems to evaluate knowledge and skills (18). The higher cognitive knowledge in medical education is based on the MCQ examinations (19). MCQs play a pivotal role in assessing thinking capabilities, critical analyzing skills, and problem-solving abilities (21, 22). MCQs examine the capability to connect concepts and analyze evidence across copious contexts. MCQs require critical thinking and analytical skills, higher-order thinking skills, which are crucial for success in higher education and professional endeavors (21, 22). Since the introduction of ChatGPT, in November 2022, few studies have been published and assessed the ChatGPT knowledge in medical sciences.

Passby et al. (24) reported that ChatGPT-3.5 obtained an overall score of 63.0%, answered the clinical questions and achieved passing marks. Duong et al. (25) investigated the capability of ChatGPT in human genetics. The overall performance was almost similar to human performance. The human response was 67.0% and “The ChatGPT response was 68.0%. Similarly, Wang et al. (26) found that the correct response rate of ChatGPT in Chinese and English questions was 54.4 and 56.9% in the first stage, and 53.8 and 67.6% in the second stage.” Suchman et al. (27) appraised the ChatGPT-3 knowledge and scored 65.0% and GPT-4 scored 62.0%. The authors highlighted its limited role in medical education. In another study, Humar et al. (28) examined the ChatGPT’s score in examination compared to working trainee students. It was found that ChatGPT answered 55.8% correctly, and performance was similar to that of first-year resident trainees.

Gilson et al. (29) found that ChatGPT obtained 44, 42, 64, and 58% scores in United States Medical Licensing Examination Step 1 and 2, respectively. The grades were significantly decreased as questions became more difficult. Similarly, Das et al. (30) observed that ChatGPT scored 80% in microbiology. In Korea, Huh et al. (31) performed an examination and compared the ChatGPT knowledge level with medical students in Korea. The overall performance of ChatGPT was lesser than the medical students. More recently, Meo and Al-Masri (32) reported that ChatGPT obtained 72% marks, a reasonable score in basic and clinical medical sciences MCQs-based examination. However, in another study, Meo and Al-Khlaiwi et al. (33) compared the Bard and ChatGPT knowledge in three different topics including endocrinology, diabetes, and diabetes technology through MCQ examination. The authors found that both ChatGPT and Bard did not achieve appropriate scores in these important subjects. ChatGPT opens multiple paths for developing knowledge with reasonings. ChatGPT provides accurate information, however, it might generate incorrect answer responses. The most probable reason is that the information is an outdated misinterpretation of complex questions, lengthy questions, and formulas (32, 33).

Similarly, Farhat et al. (34) provided valuable insights into the performance of ChatGPT-3.5, ChatGPT-4, and Bard in answering the questions. The authors reported that CHAT GPT-4 is the perfect model, highlighting its potential role in education. The literature established a benchmark for evaluating and enhancing performance in educational tasks, and its use in diverse learning environments (35). Choi (36) conducted a study using about 80 question test items from the Korean Comprehensive Basic Medical Sciences Examination, ChatGPT responded with an accuracy of 76.0%. ChatGPT was able to gather, review, and generate credible text relevant to public health and allied content. In another study, Wang et al. (37) estimated the accuracy of COVID-19 information by ChatGPT 3.5 and 4.0. The authors found that ChatGPT 3.5 and 4.0 can produce correct and appropriate COVID-19 information to a certain extent, but compared to global health organizations’ responses, gaps and deficiencies exist.

### 4.1 Implications in educational settings

The importance and implications of AI tools are swiftly increasing in various sections of society (34). In this context, this study’s findings are vital for understanding the ChatGPT knowledge for its implementation in educational settings (38). The assessment of ChatGPT knowledge could be instrumental in providing information and shaping people’s perceptions about public health allied issues such as the COVID-19 pandemic and vaccines. ChatGPT offers opportunities to enhance learning, research, assessment, and various spheres of educational settings. However, it’s essential to address ethical, quality assurance, and training considerations to maximize its benefits while mitigating potential risks (32).

### 4.2 Study strengths and limitations

Similar to other studies, this study has some strengths and limitations. The first strength is that this is a novel and interesting study that evaluates the ChatGPT knowledge on a particularly important topic of public health, infectious diseases, the COVID-19 pandemic, and its vaccines. Second, understanding the role of ChatGPT in public health, the COVID-19 pandemic and vaccine knowledge is the need of time, as ChatGPT in future may provide proper knowledge which is an acute need of the public in various fields of medical sciences including public health sciences. Third, the assessment was based on well-constructed and highly standardized MCQs. This study has some limitations. The analysis was limited by the limited size of MCQs, and the tool must be assessed in both real-world and controlled settings and its comparison with similar levels of students.

## 5 Conclusion

The knowledge of ChatGPT in public health, infectious diseases, the COVID-19 pandemic, and vaccines is adequate. The ChatGPT obtained overall satisfactory grades in all the domains of the COVID-19 pandemic-allied MCQs. The findings indicate that ChatGPT may have the potential to assist medical educationists, academicians, healthcare professionals and policymakers in providing a better understanding of infectious diseases, the COVID-19 pandemic and COVID-19 pandemic vaccines. ChatGPT could provide the latest information on public health, disease surveillance, early warnings and identify disease outbreaks. It may minimize misleading information, help to combat the pandemic, improve understanding and adherence to public health strategies and also enhance public trust. There are some challenges and gaps between the accuracy and clarity of the responses generated by ChatGPT. Similar large sample-sized studies are required to further validate the ChatGPT knowledge and effectiveness in public health, infectious diseases, the COVID-19 pandemic, and vaccines.

## Data availability statement

The raw data supporting the conclusions of this article will be available on reasonable request to corresponding author.

## Ethics statement

This study did not directly involve any animal or human participants; hence ethical approval was not required.

## Author contributions

SAM: Conceptualization, Data curation, Supervision, Writing – review & editing. MA: Data curation, Formal analysis, Investigation, Writing – original draft. MZSM: Conceptualization, Data curation, Formal analysis, Investigation, Writing – original draft. MOSM: Conceptualization, Data curation, Formal analysis, Writing – original draft. MH: Data curation, Formal analysis, Writing – original draft.

## References

[1] Salvagno M Taccone FS Gerli AG. Correction to: can artificial intelligence help for scientific writing? Crit Care. (2023) 27:99. doi: 10.1186/s13054-023-04390-0, PMID: 36890525 PMC9993712

[2] Hutson M. Could AI help you to write your next paper? Nat Res. (2022) 611:192–3. doi: 10.1038/d41586-022-03479-w, PMID: 36316468

[3] Rahman MM Watanabe Y. ChatGPT for education and research: opportunities, threats, and strategies. Appl Sci. (2023) 13:5783. doi: 10.3390/app13095783

[4] Sohail SS Farhat F Himeur Y Nadeem M Madsen DØ Singh Y. Decoding ChatGPT: a taxonomy of existing research, current challenges, and possible future directions. J King Saud Univ. (2023) 35:101675. doi: 10.1016/j.jksuci.2023.101675

[5] Hosseini M Gao CA Liebovitz DM Carvalho AM Ahmad FS Luo Y. An exploratory survey about using ChatGPT in education, healthcare, and research. medRxiv. (2023):23287979. doi: 10.1101/2023.03.31.23287979, PMID: 37796786 PMC10553335

[6] Memarian B Doleck T. ChatGPT in education: methods, potentials, and limitations. Comput Hum Behav. (2023) 1:100022. doi: 10.1016/j.chbah.2023.100022

[7] Sullivan M Kelly A McLaughlan P. ChatGPT in higher education: considerations for academic integrity and student learning. J Appl Learn Teach. (2023) 6:31–40. doi: 10.37074/jalt.2023.6.1.17

[8] Meo SA Ahmed Alqahtani S Saad Binmeather F Abdulrhman AlRasheed R Mohammed Aljedaie G Mohammed AR. Effect of environmental pollutants PM2.5, CO, O3 and NO2, on the incidence and mortality of SARS-COV-2 in largest metropolitan cities, Delhi, Mumbai and Kolkata, India. J King Saud Univ Sci. (2022) 34:101687. doi: 10.1016/j.jksus.2021.101687, PMID: 34744393 PMC8564952

[9] Hiscott J Alexandridi M Muscolini M Tassone E Palermo E Soultsioti M. The global impact of the coronavirus pandemic. Cytokine Growth Factor Rev. (2020) 53:1–9. doi: 10.1016/j.cytogfr.2020.05.010, PMID: 32487439 PMC7254014

[10] Yarlagadda H Patel MA Gupta V Bansal T Upadhyay S Shaheen N. COVID-19 vaccine challenges in developing and developed countries. Cureus. (2022) 14:e23951. doi: 10.7759/cureus.23951, PMID: 35547442 PMC9085716

[11] Kantamneni N. The impact of the COVID-19 pandemic on marginalized populations in the United States: a research agenda. J Vocat Behav. (2020) 119:103439. doi: 10.1016/j.jvb.2020.103439, PMID: 32390658 PMC7205696

[12] Bolcato M Rodriguez D Feola A Di Mizio G Bonsignore A Ciliberti R. COVID-19 pandemic and equal access to vaccines. Vaccines (Basel). (2021) 9:538. doi: 10.3390/vaccines9060538 34063863 PMC8224034

[13] Chakraborty I Maity P. COVID-19 outbreak: migration, effects on society, global environment, and prevention. Sci Total Environ. (2020) 728:138882. doi: 10.1016/j.scitotenv.2020.138882, PMID: 32335410 PMC7175860

[14] Viana J van Dorp CH Nunes A Gomes MC van Boven M Kretzschmar ME. Controlling the pandemic during the SARS-CoV-2 vaccination rollout. Nat Commun. (2021) 12:3674. doi: 10.1038/s41467-021-23938-8, PMID: 34135335 PMC8209021

[15] Ali R Sultan AS Zahid N. Evaluating the effectiveness of 'MCQ development workshop using the cognitive model framework: a pre-post study. J Pak Med Assoc. (2021) 71:119–21. doi: 10.47391/JPMA.1068, PMID: 33484534

[16] Grainger R Dai W Osborne E Kenwright D. Medical students create multiple-choice questions for learning in pathology education: a pilot study. BMC Med Educ. (2018) 18:201. doi: 10.1186/s12909-018-1312-1, PMID: 30134898 PMC6103861

[17] Kenwright D Dai W Osbourne E Gladman T Gallagher P Grainger R. “Just tell me what I need to know to pass the exam!” can active flipped learning overcome passivity? TAPS. (2017) 2:1–6. doi: 10.29060/TAPS.2017-2-1/OA1007

[18] Stringer JK Santen SA Lee E Rawls M Bailey J Richards A. Examining Bloom's taxonomy in multiple choice questions: Students' approach to questions. Med Sci Educ. (2021) 31:1311–7. doi: 10.1007/s40670-021-01305-y, PMID: 34457973 PMC8368900

[19] Vegi VAK Sudhakar PV Bhimarasetty DM Pamarthi K Edara L Kutikuppala LVS. Multiple-choice questions in assessment: perceptions of medical students from a low-resource setting. J Educ Health Promot. (2022) 11:103. doi: 10.4103/jehp.jehp_621_21, PMID: 35573621 PMC9093664

[20] Khan MU Aljarallah BM. Evaluation of modified essay questions (MEQ) and multiple-choice questions (MCQ) as a tool for assessing the cognitive skills of undergraduate medical students. Int J Health Sci (Qassim). (2011) 5:39–43. PMID: 22489228 PMC3312767

[21] Zaidi NLB Grob KL Monrad SM Kurtz JB Tai A Ahmed AZ. Pushing critical thinking skills with multiple-choice questions: does Bloom's taxonomy work? Acad Med. (2018) 93:856–9. doi: 10.1097/ACM.0000000000002087, PMID: 29215375

[22] Palmer EJ Devitt PG. Assessment of higher order cognitive skills in undergraduate education: modified essay or multiple-choice questions? Research paper. BMC Med Educ. (2007) 7:49. doi: 10.1186/1472-6920-7-49, PMID: 18045500 PMC2148038

[23] Roumeliotis KI Tselikas ND. ChatGPT, and open-AI models: a preliminary review. Fut Int. (2023) 15:192. doi: 10.3390/fi15060192

[24] Passby L Jenko N Wernham A. Performance of ChatGPT on dermatology specialty certificate examination multiple choice questions. Clin Exp Dermatol. (2023):llad197. doi: 10.1093/ced/llad197, PMID: 37264670

[25] Duong D Solomon BD. Analysis of large-language model versus human performance for genetics questions. Eur J Hum Genet. (2023). doi: 10.1038/s41431-023-01396-8, PMID: 37246194 PMC10999420

[26] Wang YM Shen HW Chen TJ. Performance of ChatGPT on the pharmacist licensing examination in Taiwan. J Chin Med Assoc. (2023) 86:653–8. doi: 10.1097/JCMA.0000000000000942, PMID: 37227901 PMC12755457

[27] Suchman K Garg S Trindade AJ. ChatGPT fails the multiple-choice American College of Gastroenterology self-assessment test. Am J Gastroenterol. (2023) 118:2280–2. doi: 10.14309/ajg.0000000000002320, PMID: 37212584

[28] Humar P Asaad M Bengur FB Nguyen V. ChatGPT is equivalent to first year plastic surgery residents: evaluation of ChatGPT on the plastic surgery in-service exam. Aesthet Surg J. (2023) 43:NP1085–9. doi: 10.1093/asj/sjad130, PMID: 37140001

[29] Gilson A Safranek CW Huang T Socrates V Chi L Taylor RA. How does ChatGPT perform on the United States medical licensing examination? The implications of large language models for medical education and knowledge assessment. JMIR Med Educ. (2023) 9:e45312. doi: 10.2196/45312, PMID: 36753318 PMC9947764

[30] Das D Kumar N Longjam LA Sinha R Deb Roy A Mondal H. Assessing the capability of ChatGPT in answering first- and second-order knowledge questions on microbiology as per competency-based medical education curriculum. Cureus. (2023) 15:e36034. doi: 10.7759/cureus.36034, PMID: 37056538 PMC10086829

[31] Huh S. Are ChatGPT’s knowledge and interpretation abilities comparable to those of medical students in Korea for taking a parasitology examination? A descriptive study. J Educ Eval Health Prof. (2023) 20:1. doi: 10.3352/jeehp.2023.20.1, PMID: 36627845 PMC9905868

[32] Meo SA Al-Masri AA Alotaibi M Meo MZS Meo MOS. ChatGPT knowledge evaluation in basic and clinical medical sciences: multiple choice question examination-based performance. Healthcare (Basel). (2023) 11:2046. doi: 10.3390/healthcare11142046, PMID: 37510487 PMC10379728

[33] Meo SA Al-Khlaiwi T AbuKhalaf AA Meo AS Klonoff DC. The scientific knowledge of bard and ChatGPT in endocrinology, diabetes, and diabetes technology: multiple-choice questions examination-based performance. J Diabetes Sci Technol. (2023):19322968231203987. doi: 10.1177/19322968231203987 37798960 PMC12035228

[34] Farhat F Chaudhry BM Nadeem M Sohail SS Madsen DØ. Evaluating large language models for the National Premedical Exam in India: comparative analysis of GPT-3.5, GPT-4, and bard. JMIR Med Educ. (2024) 10:e51523. doi: 10.2196/51523, PMID: 38381486 PMC10918540

[35] Sohail SS Madsen DØ Farhat F Alam MA. ChatGPT and vaccines: can AI chatbots boost awareness and uptake? Ann Biomed Eng. (2024) 52:446–50. doi: 10.1007/s10439-023-03305-y, PMID: 37428336

[36] Choi W. Assessment of the capacity of ChatGPT as a self-learning tool in medical pharmacology: a study using MCQs. BMC Med Educ. (2023) 23:864. doi: 10.1186/s12909-023-04832-x, PMID: 37957666 PMC10644619

[37] Wang G Gao K Liu Q Wu Y Zhang K Zhou W. Potential and limitations of ChatGPT 3.5 and 4.0 as a source of COVID-19 information: comprehensive comparative analysis of generative and authoritative information. J Med Internet Res. (2023) 25:e49771. doi: 10.2196/49771, PMID: 38096014 PMC10755661

[38] Ghosh A Maini Jindal N Gupta VK Bansal E Kaur Bajwa N Sett A. Is ChatGPT's knowledge and interpretative ability comparable to first professional MBBS (bachelor of medicine, bachelor of surgery) students of India in taking a medical biochemistry examination? Cureus. (2023) 15:e47329. doi: 10.7759/cureus.47329, PMID: 38021639 PMC10657167

